# The Kynurenine Pathway Metabolites QA and KYNA induce senescence in Bone Marrow Stem Cells through the AhR Pathway

**DOI:** 10.1093/geroni/igab046.171

**Published:** 2021-12-17

**Authors:** Dmitry Kondrikov, Ahmed Elmansi, Xing-ming Shi, Meghan McGee-Lawrence, Sadanand Fulzele, Mark Hamrick, Carlos Isales, William Hill

**Affiliations:** 1 Medical University of South Carolina, Meddical University of South Carolina, South Carolina, United States; 2 Medical University of South Carolina, Charleston, South Carolina, United States; 3 Augusta University, Augusta, Georgia, United States; 4 Medical College of Georgia, Augusta University, Augusta, Georgia, United States; 5 Medical College of Georgia, Augusta, Georgia, United States

## Abstract

Cell senescence is emerging as a critical factor in the pathophysiology of aging bone loss. We have shown that the essential amino acid tryptophan is metabolized by IDO-1 in the periphery to generate kynurenine (KYN), and that KYN can signal though the aryl hydrocarbon receptor (AhR) transcription factor pathway to inhibit osteogenesis in bone marrow MSCs via epigenetic regulation of osteogenic genes, while also upregulating osteoclastogenic transcription factors and genes driving osteoclast activity. Further, we recently showed that KYN acting via AhR inhibits MSC autophagy while inducing senescence. Here we demonstrate that KYN metabolites downstream from KYN act via the AhR signaling pathway to inhibit autophagy and induce SASP expression and drive senescence in murine and human bone marrow MSCs. We focused on two of these metabolites, quinolinic acid (QA) and kynurenic acid (KYNA) and investigated their effects on BMSC cellular function. We demonstrated that both kynurenine pathway metabolites QA and KYNA increase biomarkers for senescence including beta-galactosidase, p21/Cdkn1 and other SASPs such as PAI-1 and TIMP-2, as well as nuclear DNA damage leading to senescent markers like H2A Ser139 phosphorylation, and the accumulation of senescence-associated hetero chromatin foci (SAHF) with H3K9-me3 labeling. Then upon treatment with the AhR inhibitor 3’4’-DMF the disruption of autophagy and induction of senescent biomarkers was blocked. Like KYN, the effects of QA and KYNA were mediated through the AhR receptor. Therefore, this presents novel therapeutic targets linked to KYN metabolite signaling via AhR to prevent senescence and bone loss.

